# Supplementary material for: Macroeconomic factors affecting FDI in the African region
Source: PLoS One. 2023 Jan 23;18(1):e0280843. doi: 10.1371/journal.pone.0280843 (PMC9870099; doi:10.1371/journal.pone.0280843)
Supplement: S2 Appendix — (DOCX) [file pone.0280843.s002.docx]

**S2 Appendix. Summary of Descriptive Statistics of Variables**

| **Continents** | | | **Variables** | | | | | | | | **Continents** | | | **Variables** | | | | | | | |
| --- | --- | --- | --- | --- | --- | --- | --- | --- | --- | --- | --- | --- | --- | --- | --- | --- | --- | --- | --- | --- | --- |
|  |  |  | **FDI ($ Bn)** | | **GCI** | | **LPI** | | **IR** | |  | | | **FDI ($ Bn)** | | **GCI** | | **LPI** | | **IR** | |
| Africa | Obs. | 113 | | 113 | | 113 | | 113 | | Mauritania | | Obs. | 4 | | 4 | | 4 | | 4 | |  |
|  | Mean | 1.010 | | 3.561 | | 2.524 | | 7.377 | |  |  | Mean | 0.575 | | 3.108 | | 2.281 | | 16.964 | |  |
|  | SD | 1.780 | | 0.462 | | 0.369 | | 8.220 | |  |  | SD | 0.561 | | 0.172 | | 0.322 | | 11.560 | |  |
|  | Min. | -6.460 | | 2.597 | | 1.610 | | -33.587 | |  |  | Min. | 0.139 | | 2.940 | | 1.866 | | 5.206 | |  |
|  | Max. | 7.070 | | 4.520 | | 3.775 | | 32.768 | |  |  | Max. | 1.390 | | 3.320 | | 2.630 | | 32.768 | |  |
| Algeria | Obs. | 6 | | 6 | | 6 | | 6 | | Mauritius | | Obs. | 5 | | 5 | | 5 | | 5 | |  |
|  | Mean | 1.680 | | 3.901 | | 2.451 | | 1.762 | |  |  | Mean | 0.437 | | 4.370 | | 2.584 | | 8.022 | |  |
|  | SD | 0.315 | | 0.136 | | 0.245 | | 5.352 | |  |  | SD | 0.096 | | 0.125 | | 0.276 | | 2.581 | |  |
|  | Min. | 1.470 | | 3.720 | | 2.063 | | -6.993 | |  |  | Min. | 0.341 | | 4.200 | | 2.132 | | 6.052 | |  |
|  | Max. | 2.300 | | 4.080 | | 2.770 | | 8.326 | |  |  | Max. | 0.589 | | 4.520 | | 2.819 | | 12.525 | |  |
| Angola | Obs. | 3 | | 3 | | 3 | | 3 | | Mozambique | | Obs. | 4 | | 4 | | 4 | | 4 | |  |
|  | Mean | -2.010 | | 2.856 | | 2.279 | | -0.489 | |  |  | Mean | 2.450 | | 3.158 | | 2.375 | | 9.703 | |  |
|  | SD | 5.170 | | 0.231 | | 0.250 | | 11.154 | |  |  | SD | 2.040 | | 0.165 | | 0.208 | | 3.149 | |  |
|  | Min. | -6.460 | | 2.597 | | 2.046 | | -7.349 | |  |  | Min. | 0.417 | | 2.940 | | 2.229 | | 6.598 | |  |
|  | Max. | 3.660 | | 3.040 | | 2.543 | | 12.381 | |  |  | Max. | 5.000 | | 3.320 | | 2.684 | | 13.425 | |  |
| Benin | Obs. | 4 | | 4 | | 4 | | 4 | | Namibia | | Obs. | 5 | | 5 | | 5 | | 5 | |  |
|  | Mean | 0.152 | | 3.535 | | 2.628 | | 2.663 | |  |  | Mean | 0.561 | | 3.938 | | 2.447 | | 1.779 | |  |
|  | SD | 0.095 | | 0.143 | | 0.223 | | 3.425 | |  |  | SD | 0.305 | | 0.135 | | 0.332 | | 3.046 | |  |
|  | Min. | 0.054 | | 3.370 | | 2.428 | | -2.470 | |  |  | Min. | 0.288 | | 3.740 | | 2.020 | | -2.344 | |  |
|  | Max. | 0.282 | | 3.690 | | 2.853 | | 4.580 | |  |  | Max. | 1.040 | | 4.090 | | 2.745 | | 5.779 | |  |
| Botswana | Obs. | 4 | | 4 | | 4 | | 4 | | Nigeria | | Obs. | 6 | | 6 | | 6 | | 6 | |  |
|  | Mean | 0.256 | | 4.138 | | 2.675 | | 1.591 | |  |  | Mean | 4.680 | | 3.443 | | 2.567 | | 6.763 | |  |
|  | SD | 0.177 | | 0.111 | | 0.329 | | 6.724 | |  |  | SD | 2.290 | | 0.120 | | 0.147 | | 3.470 | |  |
|  | Min. | 0.143 | | 4.050 | | 2.318 | | -4.157 | |  |  | Min. | 0.775 | | 3.325 | | 2.396 | | 1.068 | |  |
|  | Max. | 0.515 | | 4.290 | | 3.045 | | 10.785 | |  |  | Max. | 7.070 | | 3.670 | | 2.809 | | 11.356 | |  |
| Burkina Faso | Obs. | 4 | | 4 | | 4 | | 4 | | Rwanda | | Obs. | 5 | | 5 | | 5 | | 5 | |  |
|  | Mean | 0.187 | | 3.205 | | 2.357 | | 2.186 | |  |  | Mean | 2.890 | | 4.097 | | 2.605 | | 13.230 | |  |
|  | SD | 0.181 | | 0.110 | | 0.192 | | 2.822 | |  |  | SD | 0.056 | | 0.333 | | 0.430 | | 2.602 | |  |
|  | Min. | 0.022 | | 3.070 | | 2.226 | | -0.742 | |  |  | Min. | 0.216 | | 3.563 | | 2.039 | | 11.441 | |  |
|  | Max. | 0.357 | | 3.340 | | 2.638 | | 5.996 | |  |  | Max. | 0.366 | | 4.410 | | 2.986 | | 17.683 | |  |
| Burundi | Obs. | 4 | | 4 | | 4 | | 4 | | Senegal | | Obs. | 4 | | 4 | | 4 | | 4 | |  |
|  | Mean | 0.021 | | 2.889 | | 2.187 | | 10.294 | |  |  | Mean | 0.356 | | 3.693 | | 2.576 | | 4.105 | |  |
|  | SD | 0.041 | | 0.225 | | 0.446 | | 7.652 | |  |  | SD | 0.099 | | 0.036 | | 0.226 | | 2.187 | |  |
|  | Min. | 0.000 | | 2.625 | | 1.610 | | 0.024 | |  |  | Min. | 0.272 | | 3.660 | | 2.328 | | 1.709 | |  |
|  | Max. | 0.357 | | 3.090 | | 2.566 | | 18.158 | |  |  | Max. | 0.472 | | 3.740 | | 2.863 | | 6.960 | |  |
| Congo | Obs. | 2 | | 2 | | 2 | | 2 | | Sierra Leone | | Obs. | 3 | | 3 | | 3 | | 3 | |  |
|  | Mean | 1.170 | | 2.982 | | 2.431 | | 5.406 | |  |  | Mean | 0.370 | | 2.899 | | 2.061 | | 12.489 | |  |
|  | SD | 0.336 | | 0.436 | | 0.077 | | 12.273 | |  |  | SD | 0.310 | | 0.232 | | 0.031 | | 7.221 | |  |
|  | Min. | 0.932 | | 2.674 | | 2.377 | | -3.273 | |  |  | Min. | 0.139 | | 2.716 | | 2.025 | | 7.996 | |  |
|  | Max. | 1.410 | | 3.290 | | 2.486 | | 14.084 | |  |  | Max. | 0.722 | | 3.160 | | 2.079 | | 20.819 | |  |
| Gambia | Obs. | 5 | | 5 | | 5 | | 5 | | South Africa | | Obs. | 6 | | 6 | | 6 | | 6 | |  |
|  | Mean | 0.052 | | 3.575 | | 2.425 | | 21.560 | |  |  | Mean | 4.750 | | 4.354 | | 3.541 | | 3.815 | |  |
|  | SD | 0.026 | | 0.294 | | 0.108 | | 1.538 | |  |  | SD | 1.590 | | 0.070 | | 0.154 | | 1.084 | |  |
|  | Min. | 0.023 | | 3.185 | | 2.249 | | 19.680 | |  |  | Min. | 2.220 | | 4.256 | | 3.376 | | 3.033 | |  |
|  | Max. | 0.082 | | 3.900 | | 2.518 | | 23.260 | |  |  | Max. | 6.590 | | 4.470 | | 3.775 | | 5.934 | |  |
| Kenya | Obs. | 6 | | 6 | | 6 | | 6 | | Tanzania | | Obs. | 5 | | 5 | | 5 | | 5 | |  |
|  | Mean | 0.902 | | 3.760 | | 2.750 | | 9.156 | |  |  | Mean | 1.290 | | 3.558 | | 2.532 | | 6.642 | |  |
|  | SD | 0.522 | | 0.139 | | 0.324 | | 2.533 | |  |  | SD | 0.555 | | 0.103 | | 0.343 | | 2.168 | |  |
|  | Min. | 0.178 | | 3.570 | | 2.434 | | 4.819 | |  |  | Min. | 0.582 | | 3.390 | | 2.084 | | 4.591 | |  |
|  | Max. | 1.630 | | 3.930 | | 3.331 | | 12.028 | |  |  | Max. | 1.810 | | 3.670 | | 2.990 | | 9.656 | |  |
| Lesotho | Obs. | 5 | | 5 | | 5 | | 5 | | Uganda | | Obs. | 4 | | 4 | | 4 | | 4 | |  |
|  | Mean | 0.069 | | 3.334 | | 2.243 | | 8.065 | |  |  | Mean | 0.754 | | 3.417 | | 2.732 | | 14.405 | |  |
|  | SD | 0.021 | | 0.310 | | 0.131 | | 9.845 | |  |  | SD | 0.226 | | 0.227 | | 0.250 | | 2.992 | |  |
|  | Min. | 0.041 | | 2.961 | | 2.026 | | -3.241 | |  |  | Min. | 0.544 | | 3.190 | | 2.491 | | 10.981 | |  |
|  | Max. | 0.095 | | 3.730 | | 2.373 | | 22.312 | |  |  | Max. | 1.060 | | 3.690 | | 3.043 | | 18.233 | |  |
| Liberia | Obs. | 2 | | 2 | | 2 | | 2 | | Zambia | | Obs. | 5 | | 5 | | 5 | | 5 | |  |
|  | Mean | 1.310 | | 3.460 | | 2.326 | | 8.188 | |  |  | Mean | 1.130 | | 3.479 | | 2.414 | | 4.221 | |  |
|  | SD | 1.410 | | 0.354 | | 0.173 | | 0.208 | |  |  | SD | 0.565 | | 0.287 | | 0.093 | | 2.091 | |  |
|  | Min. | 0.312 | | 3.210 | | 2.204 | | 8.040 | |  |  | Min. | 0.408 | | 3.160 | | 2.284 | | 1.715 | |  |
|  | Max. | 2.310 | | 3.710 | | 2.448 | | 8.335 | |  |  | Max. | 1.730 | | 3.860 | | 2.526 | | 6.113 | |  |
| Malawi | Obs. | 4 | | 4 | | 4 | | 4 | | Zimbabwe | | Obs. | 4 | | 4 | | 4 | | 4 | |  |
|  | Mean | 0.204 | | 3.167 | | 2.657 | | 19.786 | |  |  | Mean | 0.478 | | 3.318 | | 2.273 | | -2.930 | |  |
|  | SD | 0.269 | | 0.184 | | 0.190 | | 5.339 | |  |  | SD | 0.188 | | 0.239 | | 0.216 | | 20.535 | |  |
|  | Min. | -0.009 | | 2.968 | | 2.420 | | 12.472 | |  |  | Min. | 0.343 | | 2.982 | | 2.082 | | -33.587 | |  |
|  | Max. | 0.598 | | 3.380 | | 2.813 | | 24.625 | |  |  | Max. | 0.745 | | 3.540 | | 2.549 | | 9.744 | |  |
| Mali | Obs. | 4 | | 4 | | 4 | | 4 | |  | |  |  | |  | |  | |  | |  |
|  | Mean | 0.269 | | 3.298 | | 2.390 | | 2.204 | |  | |  |  | |  | |  | |  | |  |
|  | SD | 0.112 | | 0.201 | | 0.126 | | 2.034 | |  | |  |  | |  | |  | |  | |  |
|  | Min. | 0.144 | | 3.020 | | 2.268 | | 0.188 | |  | |  |  | |  | |  | |  | |  |
|  | Max. | 0.372 | | 3.460 | | 2.503 | | 4.019 | |  | |  |  | |  | |  | |  | |  |

Source: Authors’ creation based on data from [World Bank [1]](#_ENREF_1) and [World Economic Forum [2]](#_ENREF_2).
