# Supplementary material for: Macroeconomic factors affecting FDI in the African region
Source: PLoS One. 2023 Jan 23;18(1):e0280843. doi: 10.1371/journal.pone.0280843 (PMC9870099; doi:10.1371/journal.pone.0280843)
Supplement: S3 Appendix — (DOCX) [file pone.0280843.s003.docx]

**S3 Appendix. RE and MLR Model Results**

| **Variable** | **African Region** | **Algeria** | **Gambia** | **Kenya** | **Lesotho** | **Mauritius** | **Namibia** | **Nigeria** | **Rwanda** | **South Africa** | **Tanzania** | **Zambia** |
| --- | --- | --- | --- | --- | --- | --- | --- | --- | --- | --- | --- | --- |
|  | **lnFDI** | **lnFDI** | **lnFDI** | **lnFDI** | **lnFDI** | **lnFDI** | **lnFDI** | **lnFDI** | **lnFDI** | **lnFDI** | **lnFDI** | **lnFDI** |
|  | **RE** | **MLR** | **MLR** | **MLR** | **MLR** | **MLR** | **MLR** | **MLR** | **MLR** | **MLR** | **MLR** | **MLR** |
| LPI | 0.4660* | 0.0388 | 4.1317* | -0.5881 | 0.6530** | -1.7518* | -1.0152 | 1.1592 | 0.9020*** | -1.0997 | -3.1371 | -2.1968 |
|  | (0.2571) | (0.1240) | (0.6558) | (1.2572) | (0.0115) | (0.1842) | (0.4404) | (3.4071) | (0.0037) | (2.2717) | (0.6605) | (1.5502) |
| GCI | 0.6023 | 0.8313** | -1.4792 | 3.1456 | 1.0988*** | -2.8329* | -1.4330* | 4.8828 | -3.0705*** | -1.6343 | 12.6041 | 0.4675 |
|  | (0.3770) | (0.1743) | (0.2706) | (4.0390) | (0.0099) | (0.2395) | (0.1889) | (4.4426) | (0.0188) | (7.5294) | (2.6368) | (0.4421) |
| IR | -0.0213** | -0.0323*** | -0.0064 | -0.1203 | 0.0150*** | -0.3350** | -0.1948 | -0.0350 | -0.3863*** | 0.0036 | -0.2098 | 0.1980 |
|  | (0.0095) | (0.0032) | (0.0543) | (0.1888) | (0.0002) | (0.0258) | (0.0455) | (0.1319) | (0.0026) | (0.2833) | (0.0835) | (0.0551) |
| Constant | 16.6968*** | 17.9496*** | 13.0679 | 11.3120 | 12.7649*** | 39.4710** | 28.5090** | 2.5223 | 34.8068*** | 33.2179 | -14.6188 | 23.5505* |
|  | (1.6921) | (0.6805) | 2.1551 | (11.8809) | (0.0532) | (1.6427) | (0.7970) | (21.8830) | (0.1028) | (27.9205) | (7.2190) | (3.4747) |
| Observations | 110 | 6 | 5 | 6 | 5 | 5 | 5 | 6 | 5 | 6 | 5 | 5 |
| No. of years | 6 | 6 | 5 | 6 | 5 | 5 | 5 | 6 | 5 | 6 | 5 | 5 |
| R^2^ | 0.1821 | 0.9471 | 0.9777 | 0.2775 | 0.9999 | 0.996 | 0.9741 | 0.4246 | 1 | 0.4912 | 0.9299 | 0.9618 |

Note: *Significant at 10%, ** significant at 5%, and ***significant 1% significance level; RE denotes Random Effect model and MLR denotes Multiple Linear Regression Parentheses indicate robust standard error
