# Supplementary material for: Macroeconomic factors affecting FDI in the African region
Source: PLoS One. 2023 Jan 23;18(1):e0280843. doi: 10.1371/journal.pone.0280843 (PMC9870099; doi:10.1371/journal.pone.0280843)
Supplement: S4 Appendix — (DOCX) [file pone.0280843.s004.docx]

**S4 Appendix. Linear Fit Scatter Plot Graphs**

Source: Authors’ creation.
